# The application of blended teaching in medical practical course of clinical skills training

**DOI:** 10.1186/s12909-024-05730-6

**Published:** 2024-07-04

**Authors:** Zhicheng He, Hua Li, Lan Lu, Qiang Wang, Qingming Wu, Lili Lu

**Affiliations:** Department of Clinical Medicine, College of Medicine, Wuhan University of Science and Technology, Wuhan, Hubei P.R. China

**Keywords:** Medical education, Clinical skill training, Blended teaching, Practice course, Teaching model

## Abstract

**Background:**

Blended teaching is an effective approach that combines online and offline teaching methods, leading to improved outcomes in medical education compared to traditional offline teaching. In this study, we examined the impact of blended teaching in clinical skills training, a medical practice course.

**Methods:**

This study involved forty-eight undergraduate students studying clinical medicine in the fifth semester at Wuhan University of Science and Technology. The students were divided into two groups: the control group, which received traditional offline teaching, and the experimental group, which received hybrid teaching. Following the completion of the 4-month course, both groups underwent the Objective Structured Clinical Examination (OSCE) to evaluate their proficiency in clinical skills. Furthermore, the experimental group was given a separate questionnaire to gauge their feedback on the Blended Teaching approach.

**Results:**

Based on the OSCE scores, the experimental group outperformed the control group significantly (*P*<0.05). The questionnaire results indicated that a majority of students (54.2%, 3.71 ± 1.06) believed that blended teaching is superior to traditional offline teaching, and a significant number of students (58.3%, 3.79 ± 1.15) expressed their willingness to adopt blended teaching in other courses. Furthermore, students in the experimental group displayed varying levels of interest in different teaching contents, with emergency medicine (79.2%), internal medicine (70.8%), and surgery (66.7%) being the most popular among them.

**Conclusions:**

This research demonstrates for the first time that blended teaching can achieve a good pedagogical effectiveness in the medical practice course, clinical skills training and practice. Moreover, in different teaching contents, the teaching effects are different. In the content of Emergency Medicine and Surgery, which is more attractive to students, the application of blended teaching could result in a better pedagogical outcome than other contents.

## Introduction

The education system for clinical medicine students in China primarily follows the “5 + 3” model, with some variations such as the 8-year program. In the “5 + 3” model, students undergo a five-year undergraduate program to get a bachelor degree and then followed by three-year standardized residency training [1]. Undergraduate education can be divided into three parts: theoretical learning, apprenticeships, and internships. At Wuhan University of Science and Technology (WUST), the undergraduate clinical medical education program follows a unique 2.5 + 2.5 model. In this model, the first 2.5 years are dedicated to studying general courses and basic medical courses at the university. The subsequent 2.5 years are then devoted to completing clinical courses and clinical internships in affiliated hospitals.

In the career of clinical medical students, the acquisition of clinical skills is crucial for demonstrating competence in clinical practice. Before the internship stage, medical students are typically expected to master fundamental clinical skills such as physical examination, cardiopulmonary resuscitation (CPR), major puncture operations, and basic surgical operations. These skills will play an essential role in their future careers. For instance, proficient physical examinations can expedite the treatment process for patients with acute and serious illnesses, as well as guide doctors in conducting other necessary examinations promptly. This not only reduces the financial burden on patients but also improves the allocation of medical resources [2]. Additionally, regardless of the department they work in, it is imperative for medical students to master CPR and be able to apply it in emergencies [3]. According to this requirement, WUST has introduced the course “Clinical Skills Training and Practice” in the fifth semester to cultivate students’ basic clinical skills before they enter the affiliated hospitals.

With the rapid development of information technology, the traditional offline medical teaching mode alone is no longer sufficient to meet the evolving needs of medical education in this era. Initially, online medical education was primarily limited to recording and broadcasting courses, often spread through tapes and CDs. This traditional model, however, only catered to basic teaching needs with limited interactivity and feedback. The emergence of internet technologies has paved the way for innovative teaching methods such as online instruction and virtual simulations to gain popularity. These advancements have made teachers more engaging to students and facilitated increased feedback [4, 5]. Moreover, the research on the utilization of ChatGPT in medical education reminds us that the evolution of medical education will progress alongside advancements in science and technology [6]. Changes in educational methods are influenced not only by technological advancements but also by shifts in students’ intrinsic needs. In today’s information-rich environment, traditional teaching methods centered around knowledge transfer alone fall short in meeting students’ requirements. The progress in technology has expanded the possibilities for educational approaches, including the flipped classroom model, project-based learning, and differentiated instruction. These innovations enable educators to focus on enhancing students’ learning experiences, increasing their interest and engagement, catering to their diverse and personalized learning preferences, and ensuring fair and inclusive access to education for a broader audience.

Traditional clinical skills training typically involves a structured presentation by the teacher, followed by the student’s practice under supervision [7]. However, the COVID-19 pandemic has boosted the development of online courses, such as ‘Clinical Skills Training and Practice’ in WUST. Multiple studies demonstrate that online medical education during this period has yielded unexpected advancements and potential [8–10]. In the past three years, WUST’s online “Clinical Skills Training and Practice” course has demonstrated promising results in improving pedagogical effectiveness. This prompts us to consider whether blending online education with traditional offline teaching (TOT) could be a better option. Blended teaching (BT), which combines online and offline methods, has been used in medical education since 1990 [11, 12]. A meta-analysis comparing BT and TOT in medical education indicates that BT has superior pedagogical effectiveness [13].

During the transition from being a medical student to becoming a doctor, students need to take medical practice courses to enhance their understanding and application of theoretical knowledge [14]. Among these courses, clinical skills training is particularly challenging and crucial due to its practical nature. While there has been limited research on the use of BT in clinical skills training.

This study, conducted at the Clinical Skills Training Center of Wuhan University of Science and Technology, aims to investigate the effectiveness of BT in clinical skills training. The participants of this study were undergraduate students majoring in clinical medicine in their fifth semester. The researchers implemented either BT or TOT in their ‘Clinical Skills Training and Practice’ course. The teaching effectiveness was evaluated using Objective Structured Clinical Examination (OSCE) scores and questionnaires. It is hypothesized that students who receive BT will achieve higher OSCE scores and report a more positive teaching experience and effectiveness in the questionnaire.

## Methods

### Subjects

The study utilized a prospective randomized controlled design and received approval from the Ethics Committee of Wuhan University of Science and Technology (Dossier number 2022151). Sample size was computed with the aim of 0.85 power value, predicated on an effect size of 0.9 and a margin of error set at 0.05. A minimum of 19 participants per group was calculated using PASS 15, resulting in the recruitment of a total of 38 undergraduate students. To address the potential issue of sample dropout during project implementation, the sample size was increased to 48 students. 48 students were recruited based on predefined inclusion criteria from the total 248 third-year undergraduate students from the Department of Clinical Medicine at WUST. The inclusion criteria included: (1) proficient communication and comprehension skills, (2) consistent attendance without absenteeism or truancy, and (3) a positive attitude toward learning. Exclusion criteria comprised: (1) refusal to participate, (2) class absence, (3) failure to complete the final test, and (4) incomplete questionnaire responses. The study emphasized voluntary participation, allowing participants to withdraw at any time without providing a reason. We employed a random digital method to create a set of identification numbers, which were subsequently placed in a box and shuffled. Participants then selected codes from the box to determine their assignment to either the experimental Group A (*n* = 24) or the control Group B (*n* = 24). The random allocation sequence was generated using IBM SPSS Statistics 27. The study was conducted from September 2022 to December 2022. Prior to the commencement of the study, none of the participants had undergone any clinical skills training.

### Study design

According to the WUST clinical medicine cultivation program, the course “Clinical Skills Training and Practice” is conducted in the fifth semester. Both groups of students followed the same syllabus and were taught and assessed by the same teaching team. The objectives of this course include gaining theoretical knowledge of various clinical operations and achieving proficiency in performing CPR, the four major puncture operations (thoracentesis, lumbar, myelopuncture, and peritoneal puncture), physical examination, and basic surgical operations (Disinfect & Draping, Donning & Taking off Surgical Gowns, and Incision & Suturing). All faculty members involved in this course are part of the Department of Clinical Medicine, holding both medical practitioner and teaching certificates, and possessing extensive teaching skills and clinical experience. Offline lessons took place at WUST’s Clinical Skills Training Center. The designated textbook for this course is ‘Clinical Skills Training and Practice’ [15]. The course consists of 144 periods and lasts approximately 4 months.

### Interventions

**Fig. 1:**
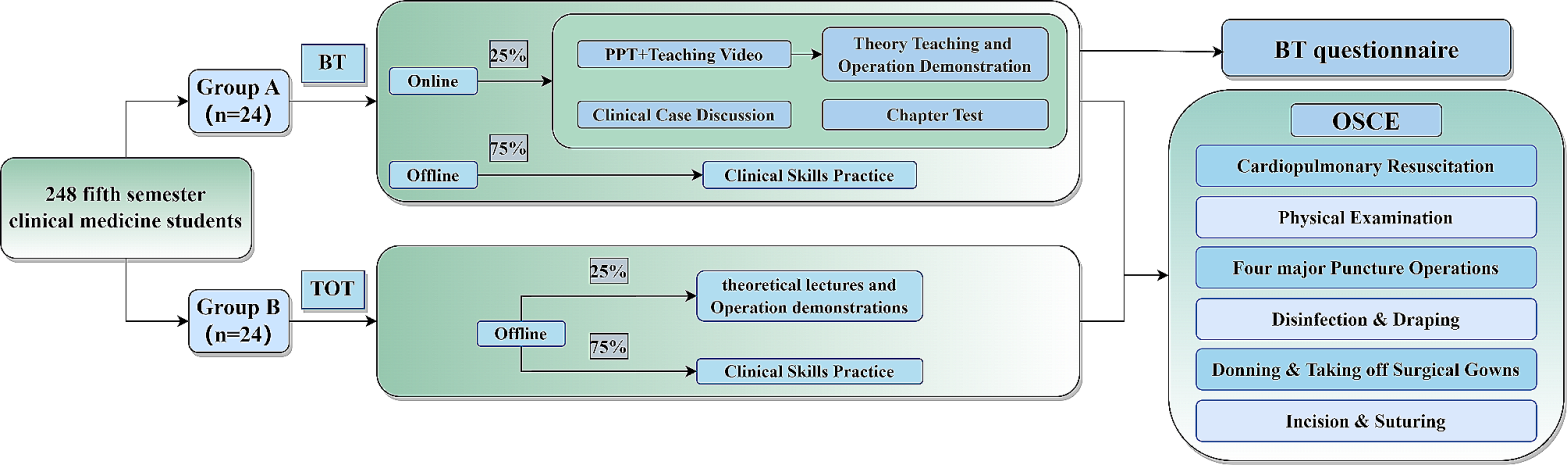
An overview of the course design: From 248 fifth-semester clinical medicine students, 48 students were randomly selected and divided into Group A and Group B. Group A adopted BT, and Group B adopted TOT. After 4 months of teaching, both of the two groups took OSCE but only Group A took the questionnaire BT: Blended Teaching; TOT: Traditional Offline Teaching

Group A utilized the online course called “Clinical Skills Training and Practice” available on the University Open Online Courses (UOOC) [16]. The course is divided into five clinical modules: internal medicine, surgery, gynecology, pediatrics, and emergency medicine. Each module consists of theoretical lecture videos, standardized operation demonstration videos, PPT resources, as well as supporting exercises and tests. The course platform also provides a discussion and exchange board for teachers and students to interact and discuss topics online. The online teaching component constitutes 25% of the total class hours (Fig. 1).

**Fig. 2:**
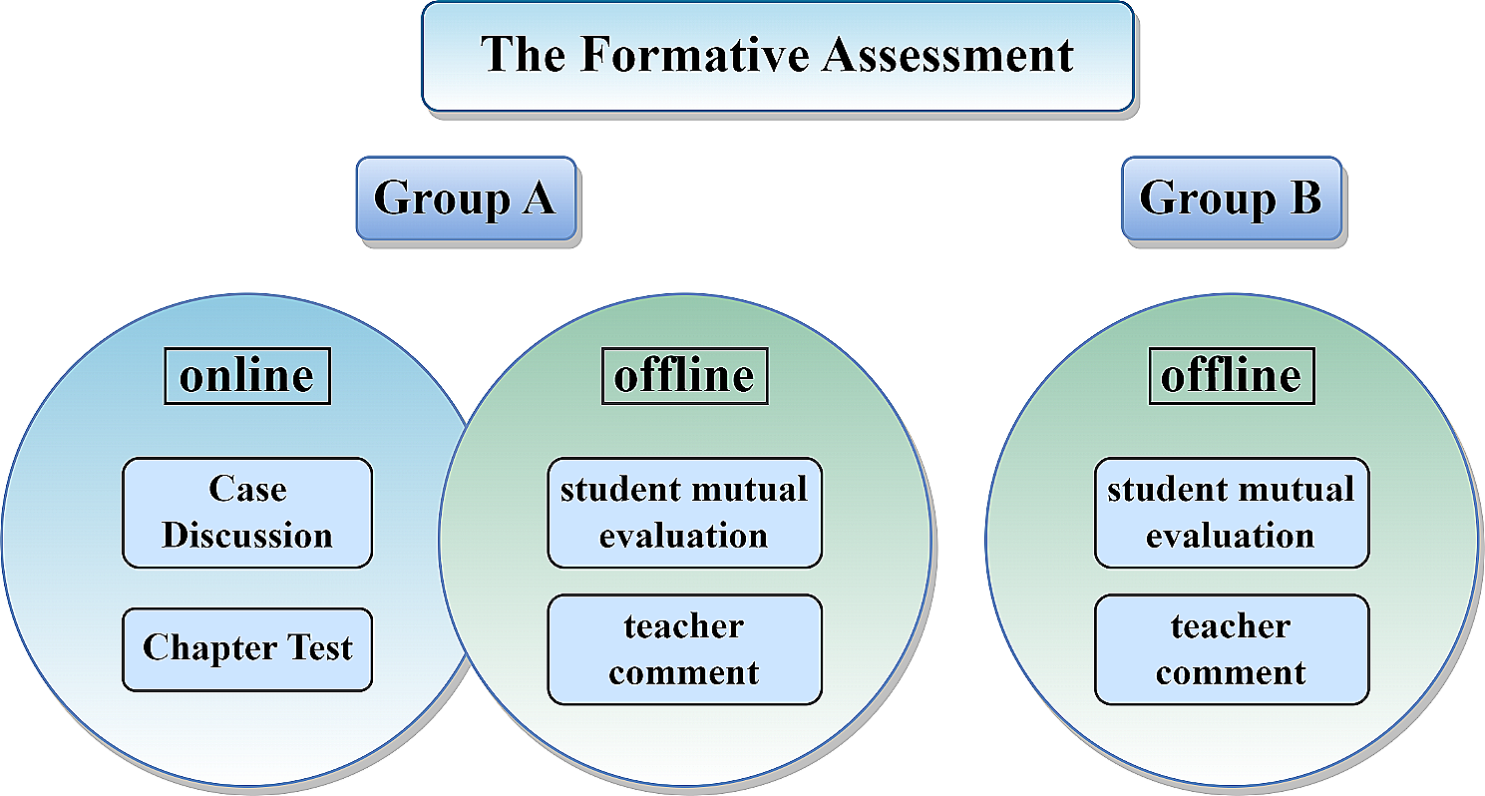
The formative assessment of Group A and Group B

Before each offline class, the teacher publishes the teaching content on the platform. Students access the platform using electronic devices and independently learn the relevant material. Through platform data, teachers can monitor and adjust the offline teaching content based on students’ progress. For skills that students have mastered well, teachers will primarily guide students to practice independently during offline teaching. For skills with weak mastery data, teachers will initially emphasize the key points of skill operation and provide demonstrations during offline teaching. The approach of targeting weak areas will be more focused, avoiding redundant explanations of basic content, and offering students more chances for self-practice. Instead of traditional lectures and demonstrations, teachers guide students in practical exercises during offline classes and enhance learning through formative evaluations such as group evaluations and teacher feedback (Fig. 2). After the offline classes, students return to the online platform to complete tests and assignments for each chapter, reinforcing their understanding of the acquired skills. If students encounter any difficulties, they can communicate with the teacher through the online course platform’s discussion area, ensuring timely teacher-student communication. Additionally, the course team teachers utilize the discussion area of the online platform to provide high-level clinical thinking training content, such as case analysis, to cater to the individualized learning needs of students at higher levels. The specific teaching process is depicted in Fig. 1.

Group B students adopt the TOT model, which includes theoretical teaching and demonstration conducted by the teacher (25% of class time) followed by practical exercises by the students (75% of class time) (Fig. 1). Additionally, student mutual evaluation and teacher comments are used to conduct formative evaluation of students’ learning effects (Fig. 2).

### Data collection

After the course, both groups of students underwent offline OSCE assessments at the WUST Clinical Skills Training Center. These assessments were conducted by the same group of examiners. The OSCE assessment consisted of 6 examination stations, namely: physical examination, cardiopulmonary resuscitation, four major puncture operations, donning & taking off surgical gowns, disinfection & draping, and incision & suturing (Fig. 1).

We designed a questionnaire for students in Group A who adopted BT. We used Alpha to calculate intra-group consistency and reliability. The alpha value of the BT questionnaire in Group A is 0.941, indicating that the questionnaire meets the required reliability. After the OSCE, the teacher distributed an anonymous questionnaire to the students in Group A (Fig. 1). The questionnaire included two basic pieces of information about the subjects’ age and gender, 11 scale questions, 1 multiple-choice question, and 1 open-ended question. The question design is based on a Likert scale (the scale ranges from 1 to 5, indicating the degree from strongly disagree to strongly agree). We considered a score ≥ 4 as an agreement.

The primary outcome of this study was to evaluate the scores of OSCE at the end of the course for both groups of students. Additionally, the results of the questionnaire were considered as a secondary outcome.

### Statistical analyses

We used the Mac 2019 version of Microsoft Excel to collect all the OSCE score data and the BT questionnaire data. IBM SPSS Statistics 27 was used to test the normality and homogeneity of variance between groups A and B. Continuous variables with normal distribution were presented as mean ± standard deviation (SD); non-normal variables were reported as median (interquartile range). Suppose the data matched the normal distribution the independent samples t-test was used, if not the Mann-Whitney U-test was used. Frequency analysis was conducted to analyze the rate of students’ agreement with each question in the BT questionnaire as reflected in the count data and expressed as a percentage (%). *P* < 0.050 determined that it was statistically significant.

## Results

### Participants’ demographic data

The demographic data of Groups A and B are presented in Table 1. This study comprised a total of 48 students, with 24 students in Group A and 24 students in Group B. The average age of the students in Group A was 20.08 ± 0.65, while in Group B it was 21.33 ± 0.92. The male/female ratio in Group A was 9/15 and in Group B was 12/12.

**Table 1:** Demographic data analysis

	Group A (*n* = 24)	Group B (*n* = 24)
Characteristics	Mean ± SD/ N(%)	Mean ± SD/ N(%)
Age (year)	20.08 ± 0.65	21.33 ± 0.92
Gender(male/female)	9/15	12/12

Age is expressed as the means ± SD. Group A, BT. Group B, TOT

### Results of OSCE

As demonstrated in Table 2, the normality of the data was assessed using the Shapiro-Wilk Test. It is important to highlight that the *P* value for group A in the total score item is less than 0.05, indicating a lack of normal distribution characteristics. Notably, in studies with small sample sizes (*n* < 50), meeting the criteria for data normality might be challenging. However, if the absolute value of Skewness is below 10 and the absolute value of Kurtosis is below 3, it is acceptable to proceed with corresponding statistical analyses as if the data is normality. Subsequently, based on the outcomes of the data analysis, appropriate statistical methods are applied to different types of data. The OSCE scores are shown in Table 3. Significant differences were observed in various skills between Group A and Group B. These skills include cardiopulmonary resuscitation (92.50(4.00) vs. 86.00(3.50), *P* < 0.050), physical examination (90.04 ± 3.09 vs. 63.83 ± 7.03, *P* < 0.050), four major puncture operations (77.21 ± 8.99 vs. 71.17 ± 6.42, *P* < 0.050), disinfection & draping (82.79 ± 4.03 vs. 61.42 ± 12.48, *P* < 0.050), donning & taking off surgical gowns (84.00(5.75) vs. 78.00(9.00), *P* < 0.050), incision & suturing (68.42 ± 5.26 vs. 62.79 ± 8.30, *P* < 0.050) and total score (82.13 ± 3.36 vs. 70.00 ± 5.77, *P* < 0.050). These results indicate that Group A achieved higher average scores than Group B in all evaluated items at the significance level of 0.05.

**Table 2:** Normality test of students’ OSCE

Content	Group	Shapiro-Wilk Test				Normal Distribution?	Analytical Method
		Skewness	Kurtosis	W-value	*P*-value		
Cardiopulmonary Resuscitation	A	-2.172	7.107	0.799	< 0.001*	No	Mann-Whitney U-test
	B	-1.288	2.370	0.880	0.008*	No	Mann-Whitney U-test
Physical Examination	A	0.161	-0.447	0.953	0.314	Yes	Independent samples t-test
	B	-0.076	2.063	0.938	0.150	Yes	Independent samples t-test
Four Major Puncture Operations	A	-0.062	-0.693	0.984	0.954	Yes	Independent samples t-test
	B	-0.323	-0.760	0.954	0.330	Yes	Independent samples t-test
Disinfection & Draping	A	-0.811	0.339	0.918	0.053	Yes	Independent samples t-test
	B	-0.290	0.528	0.973	0.748	Yes	Independent samples t-test
Donning & Taking off Surgical Gowns	A	-1.890	5.030	0.832	0.001*	No	Mann-Whitney U-test
	B	-1.070	0.333	0.868	0.005*	No	Mann-Whitney U-test
Incision & Suturing	A	1.012	1.407	0.937	0.140	Yes	Independent samples t-test
	B	-0.242	1.634	0.950	0.266	Yes	Independent samples t-test
Total Score	A	-1.255	2.416	0.911	0.038*	No	Independent samples t-test
	B	-0.536	-0.286	0.962	0.489	Yes	Independent samples t-test

It indicates that there is a difference below the significance level of 0.05

**Table 3:** Results of students’ OSCE

Content	Group A	Group B	T/Z	*p*-value
Cardiopulmonary Resuscitation	92.50(4.00)	86.00(3.50)	-4.835	<0.001*
Physical Examination	90.04 ± 3.09	63.83 ± 7.03	16.732	<0.001*
Four Major Puncture Operations	77.21 ± 8.99	71.17 ± 6.42	2.680	0.010*
Disinfection & Draping	82.79 ± 4.03	61.42 ± 12.48	7.985	<0.001*
Donning & Taking off Surgical Gowns	84.00(5.75)	78.00(9.00)	-3.467	0.001*
Incision & Suturing	68.42 ± 5.26	62.79 ± 8.30	2.804	0.007*
Total Score	82.13 ± 3.36	70.00 ± 5.77	8.897	<0.001*

It indicates that there is a difference below the significance level of 0.05

### Perspectives survey about online and offline blended teaching mode

Table 4 presents the experiences and opinions of students in Group A regarding BT. The questionnaire was designed with questions categorized into four dimensions: course experience, learning effect, teaching evaluation, and overall evaluation. By analyzing the responses to questions 1–4, we can assess the impact of students’ course experience. Among the students who adopted BT, a higher number of students reported an improved course experience. Specifically, the model aided in understanding the theoretical knowledge of clinical skill operations (70.8%, 4.04 ± 1.10) and facilitated faster independent learning of these operations (70.8%, 4.04 ± 1.17). Additionally, it promoted the speed of mastering skills (66.6%, 3.92 ± 1.22) without significantly increasing the learning burden, as observed under good teaching effects (70.8%, 2.79 ± 1.15). Questions 5–7 aimed to assess student learning effectiveness. The majority of students expressed that BT helped prepare for OSCE exams (66.7%, 3.83 ± 1.18), promoting self-directed learning that is not bound by time and space (62.5%, 3.83 ± 1.14), and increasing their interest in the learning process (58.3%, 3.79 ± 1.08). Students’ evaluation of teaching under BT can be assessed using questions 8–9. The majority of students expressed that both online instruction (62.5%, 3.75 ± 1.13) and offline instruction (70.9%, 3.96 ± 1.14) in BT were effective in achieving the desired outcomes and objectives. The students’ overall assessment of BT is reflected in questions 10–11. More than half of the students (54.2%, 3.71 ± 1.06) felt that BT was better than TOT, and a higher proportion of the students (58.3%, 3.79 ± 1.15) expressed their willingness to implement BT into other medical skills training. Furthermore, question 12 revealed the students’ interest in each part of the teaching content, indicating that emergency medicine (79.2%), internal medicine (70.8%), and surgery (66.7%) were the most popular choices.

**Table 4:** The method of questionnaire was designed to investigate the feelings of BT mode in Group A

Content	5	4	3	2	1	Average score
Q1: Online and offline blended teaching mode helps to understand the key points and precautions of clinical skills operation	11/24 (45.8%)	6/24 (25.0%)	5/24 (20.8%)	1/24 (4.2%)	1/24 (4.2%)	4.04 ± 1.10
Q2: Online and offline blended teaching mode helps to complete the skills independently	12/24 (50.0%)	5/24 (20.8%)	4/24 (16.7)	2/24 (8.3%)	1/24 (4.2%)	4.04 ± 1.17
Q3: Online and offline blended teaching mode helps to facilitate faster mastery of skills operation	11/24 (45.8%)	5/24 (20.8%)	4/24 (16.7)	3/24 (12.5%)	1/24 (4.2%)	3.92 ± 1.22
Q4: Online and offline blended teaching mode increases the burden of hands-on skills learning	2/24 (8.3%)	5/24 (20.8%)	6/24 (25.0%)	8/24 (33.3%)	3/24 (12.5%)	2.79 ± 1.15
Q5: Online and offline blended teaching mode is helpful for OSCE skills exams	9/24 (37.5%)	7/24 (29.2%)	4/24 (16.7)	3/24 (12.5%)	1/24 (4.2%)	3.83 ± 1.18
Q6: Online and offline blended teaching mode is helpful for self-directed learning (not limited by time and space)	9/24 (37.5%)	6/24 (25.0%)	6/24 (25.0%)	2/24 (8.3%)	1/24 (4.2%)	3.83 ± 1.14
Q7: Online and offline blended teaching mode is helpful in enhancing interest in learning	8/24 (33.3%)	6/24 (25.0%)	8/24 (33.3%)	1/24 (4.2%)	1/24 (4.2%)	3.79 ± 1.08
Q8: How satisfied are you with the theory taught and the operational demonstrations in the online course?	8/24 (33.3%)	7/24 (29.2%)	6/24 (25.0%)	2/24 (8.3%)	1/24 (4.2%)	3.75 ± 1.13
Q9: How satisfied are you with the operational demonstrations and training in the offline program?	10/24 (41.7%)	7/24 (29.2%)	4/24 (16.7)	2/24 (8.3%)	1/24 (4.2%)	3.96 ± 1.14
Q10: Whether the online-offline blended teaching model (BT) is better than the traditional offline teaching model (TOTM).	7/24 (29.2%)	6/24 (25.0%)	9/24 (37.5%)	1/24 (4.2%)	1/24 (4.2%)	3.71 ± 1.06
Q11: Would you like to adopt a blended online and offline teaching model for other medical skills training projects?	9/24 (37.5%)	5/24 (20.8%)	7/24 (29.2%)	2/24 (8.3%)	1/24 (4.2%)	3.79 ± 1.15
	emergency Medicine	Internal Medicine	surgery	Gynecology	pedology	
Q12: What part of the skills training did you find most helpful or interesting? (multiple choice)	19/24 (79.2%)	17/24 (70.8%)	16/24 (66.7%)	9/24 (37.5%)	10/24 (41.7%)	
Q13: Do you have any thoughts or suggestions for Online and offline blended teaching mode?						

point Likert responses (5 = strongly agree, 1 = strongly disagree) collected via Score table. Scores were categorized where ≥ 4 was defined as consent

## Discussion

Clinical skills training in clinical practice courses is characterized by a high degree of practicality and the requirement for more practice time. The TOT model is commonly used, where instructors teach the theory and demonstrate the skills, followed by students practicing on their own. However, this model often limits the duration of students’ practical exercises, which is not beneficial to the training of clinical skills. The present study aims to assess the potential of BT in practice course by integrating online courses with offline practice, developing a BT course that meets pedagogical requirements, and evaluating its teaching effectiveness in different clinical skills. The research findings indicate that students in Group A, who adopted BT, performed better overall in OSCE compared to Group B, who followed the TOT model. Moreover, the results of the questionnaire revealed that Group A students had a positive learning experience and perceived the course to be more effective in terms of pedagogy.

The OSCE is widely recognized as an effective way to judge students’ mastery of clinical skills for formative and summative purposes [17]. In terms of OSCE scores, students in Group A outperformed those in Group B in both the overall score and each individual item. The differences between all items were statistically significant. When comparing the average scores of each item between the two groups, it can be observed that Group A showed varying levels of improvement in different assessment items. The performance difference between groups A and B was more obvious in the two items of physical examination and Disinfection & Draping compared to the other items. This suggests that although BT demonstrated better teaching effectiveness overall, its strengths vary across different types of items. These two items stand out due to their extensive content but relatively simple operation. With the use of the online platform in BT, students have the opportunity to repeatedly learn and become more proficient in these operations. However, when faced with tasks that require more offline practice, such as CPR and the four major puncture operations, the performance improvement is not as significant as observed in the two aforementioned items. That means online teaching cannot fully substitute offline teaching, especially when it comes to highly practical teaching content. However, online course platforms can be utilized to enhance teaching content, broaden teaching activities, and compensate for the limitations of traditional offline teaching. The results of the questionnaire in Group A revealed that students demonstrated a great interest interest in first aid, internal medicine, and surgery skills. Additionally, Group A achieved higher scores in the OSCE at the CPR site (Emergency Medicine) and the Basic Surgical Skills-related site (Surgery). These findings indicate that when students are presented with more engaging study materials, their motivation to learn is enhanced, leading to improved learning outcomes driven by higher levels of initiative [18]. Therefore, in the next stage of course construction, it is crucial to explore the development of more course content that can effectively enhance students’ interest in learning.

In contrast to this study, much of the current research on the use of BT in clinical skills education tends to concentrate on specific skills or skill types. Amy L Halverson’s research, for example, delves into surgical skills. The findings of Halverson’s study indicate that BT has a beneficial impact on surgical skills training for rural physicians, aligning with the outcomes of our study. Nevertheless, unlike the present research, Halverson’s study relied solely on questionnaires for drawing conclusions and lacked objective evaluation metrics [19]. More studies are focusing on evaluating the effectiveness of the BT model in CPR training due to the broad audience it caters to, which includes both medical and non-medical professionals. A study conducted on 832 non-medical professional persons in Taiwan revealed that the BT model was superior to TOT [20]. Additionally, research on the application of the BT model in CPR training for underage students demonstrated a significant increase in students’ willingness to intervene during a cardiac arrest, from 56.9 to 93.1% post-course [21]. These findings highlight the positive impact of the BT model on students’ self-confidence and overall teaching outcomes. Our study further supports these results, as the group A trained with the BT model performed notably better in the OSCE at the CPR site. This study innovatively applied the BT model to various types of clinical skills training, comparing its effects with the TOT model across different skill items. Moreover, this research not only examined the differences in application effects between the two teaching models on the same skill items but also compared the differences in teaching effectiveness improvement after applying the BT model among different skill items. The findings offer a more comprehensive theoretical foundation for application of the BT teaching model in clinical skills practice courses.

In the design of the course, we offer a wealth of clinical case materials on the online course platform. These materials are available for students who are eager to learn. Our goal is to foster students’ advanced abilities through the use of relevant cases or scenarios, which can enhance their coping skills and their ability to handle emergencies [22]. Our study has shown that students in Group A demonstrate higher performance in practical projects like CPR, which require hands-on experience, through online situational clinical case training. This training method allows students to go beyond simply acquiring visual information and instead encourages them to analyze, process, and integrate the visual information. As a result, students can achieve a deeper understanding of the knowledge points, progressing from the lower levels of Bloom’s taxonomy (memorization and comprehension) to higher levels such as analysis, application, and judgment. This approach greatly enhances the effectiveness of learning [23]. According to several studies, virtual simulation has been found to be more effective in promoting the learning of skills compared to teaching theoretical knowledge alone [24]. Therefore, in future designs of BT, we propose incorporating virtual simulation teaching into the online platform. This addition aims to address the limitations of the online platform in practical training and enhance the overall learning experience [25].

The blend of online courses with the traditional TOT model can offer teachers a more personalized teaching environment and timely feedback. The online education platform enables real-time observation and regulation of students’ learning progress, allowing for dynamic adjustments in offline teaching content and methods to better achieve pedagogical goals. Furthermore, we designed a chapter test in the online course. According to Kromann, the inclusion of testing in clinical skills training can be effective in improving the effectiveness of learning [26]. In this study, we observed that teachers can effectively assess students’ understanding of this particular aspect of the theory through chapter tests. This allows them to provide targeted guidance and reinforcement for students’ weaker areas in the offline course. Such feedback evaluation, developed during the teaching process, plays a crucial role in improving teaching effectiveness due to its timeliness and relevance. In future course designs, we plan to incorporate various forms of accompanying tests in both online and offline sessions to further enhance formative evaluation and teaching effectiveness.

For students, blending the online course with the offline course can provide the advantages of being more accessible and flexible in terms of time and location. It has been claimed that students can arrange their learning according to their own schedule and rhythm through the online platform in the BT model [13]. A similar phenomenon was observed in our study. For instance, before each offline teaching session or OSCE, there was a noticeable increase in students accessing online platforms. This trend indicates that students are using online platforms to align with their learning or revision strategies. In the online course, we have also introduced a discussion board where the instructor posts clinical case information and related questions. This board serves as a platform for students to actively participate in discussions and answer the questions posed by the instructor. The instructor then provides feedback on the student’s answers. This interactive communication method helps to reinforce the students’ clinical knowledge and skills, while also training them to develop their initial clinical thinking skills. Meanwhile, it also can effectively promote student participation in this course. It has been reported that greater student engagement in courses can increase their positive experience of the course and ultimately improve the effectiveness of the instruction [27]. However, in the BT model, students are required to possess advanced self-management skills and be familiar with online teaching platforms. Therefore, it is essential to integrate suitable learning monitoring tools and provide adequate training as part of the teaching process [28, 29].

In the analysis of the questionnaire, we also noticed that students were slightly more satisfied with offline education (70.9%) than with online education (62.5%). This reminds us that offline teaching still holds its irreplaceability compared to online teaching. For example, face-to-face communication in offline teaching fosters a closer emotional connection between teachers and students. It allows for more intuitive guidance in developing students’ skills and provides faster feedback [30]. Given the practical nature of clinical skills courses, it is reasonable to conclude that online teaching cannot fully replace offline teaching. However, our research indicates that a combination of online and offline instruction can produce a synergistic effect. The online component of the course expands teaching resources and diversifies teaching methods, while also overcoming time and space constraints and promoting independent learning. On the other hand, the offline component allows teachers to provide personalized face-to-face guidance promptly. By combining these two approaches, we can achieve improved pedagogical effectiveness by leveraging their complementary advantages.

Like all educational research articles, this study has some limitations. Firstly, the sample size in this study is relatively small, which may result in a larger margin of error. Therefore, in our future studies, we plan to increase the sample size to reduce the potential bias caused by the small sample. Additionally, the limited number of clinical skills items included in this research may not provide a comprehensive evaluation of the effectiveness of BT in various clinical skills teaching. In future research, we will incorporate more measures to assess the learning outcomes of students’ clinical skills. This will involve collecting scores from graduation operation examinations and licensing examinations to objectively evaluate students’ mastery of clinical skills. Additionally, we will enhance curriculum development by integrating more clinical skills teaching programs into the BT model. This will allow for a more comprehensive evaluation of the BT model’s effectiveness in training various clinical skills programs. The questionnaire used in this study may have limitations in evaluating the teaching effect of BT due to its subjective nature. It is more suitable for assessing students’ subjective perceptions of the BT teaching model. Future research will aim to enhance the questionnaire design to better capture the subjective experiences of both teachers and students.

The development of the times has resulted in significant changes in medical education. As educators, it is important for us to actively explore new teaching modes and methods to enhance students’ learning experiences and outcomes. This will enable us to better cultivate medical students to meet the demands of the modern era. In conclusion, the results of this research indicate that students adopting BT are better in clinical skills training than those adopting TOT. And then, BT was better at teaching content-rich but easy-to-do items (physical examination and disinfection & draping) than practice-demanding items. Finally, students adopting BT will have better pedagogical outcomes in the more interesting items (emergency medicine and surgery). The application of BT in clinical skills training has demonstrated its potential in this study, leading us to believe that applying BT to other medical skills training and courses could yield unexpected benefits. In the future, we plan to develop more courses using blended teaching to cater to the needs of the new generation of clinical medical students.

## Data Availability

The datasets used and analysed during the current study available from the corresponding author on reasonable request.
